# Comparative analysis of the impact of 40 adenovirus types on dendritic cell activation and CD8^+^ T cell proliferation capacity for the identification of favorable immunization vector candidates

**DOI:** 10.3389/fimmu.2023.1286622

**Published:** 2023-10-17

**Authors:** Xiaoyan Wang, Mario Hetzel, Wenli Zhang, Anja Ehrhardt, Wibke Bayer

**Affiliations:** ^1^ Institute for Virology, University Hospital Essen, University Duisburg-Essen, Essen, Germany; ^2^ Virology and Microbiology, Center for Biomedical Education and Research (ZBAF), Faculty of Health, Witten/Herdecke University, Witten, Germany

**Keywords:** human adenovirus (HAdV), adenoviral vector, adenovirus-based immunization, CD8 T cell response, CD8+ T cell response, T cell response, immunogenicity

## Abstract

For the development of new adenovirus (AdV)-based vectors, it is important to understand differences in immunogenicity. In a side-by-side *in vitro* analysis, we evaluated the effect of 40 AdV types covering human AdV (HAdV) species A through G on the expression of 11 activation markers and the secretion of 12 cytokines by AdV-transduced dendritic cells, and the effect on CD8^+^ T cell proliferation capacity. We found that the expression of activation markers and cytokines differed widely between the different HAdV types, and many types were able to significantly impair the proliferation capacity of CD8^+^ T cells. Univariate and multivariate regression analyses suggested an important role of type I interferons in mediating this suppression of CD8^+^ T cells, which we confirmed experimentally in a proliferation assay using a type I interferon receptor blocking antibody. Using Bayesian statistics, we calculated a prediction model that suggests HAdV types HAdV-C1, -D8, -B7, -F41, -D33, -C2, -A31, -B3 and -D65 as the most favorable candidates for vaccine vector development.

## Introduction

The virus family Adenoviridae comprises a large number of animal and human adenoviruses (AdV), with the International Committee on Taxonomy of Viruses listing 54 human AdV (HAdV) types in the genus Mastadenovirus and 64 non-human primate AdV types (1), with the non-human primate AdV types belonging to the subgenus Simian Mastadenovirus or to the subgenus Human Mastadenovirus due to their close phylogenetic relationship. The Human Adenovirus Working Group, on the other hand, even assigns more than 100 HAdV types (2). HAdV are grouped into seven different species A through G, and can result in mainly respiratory, gastrointestinal, ocular or genitourinary infections.

AdVs are popular vectors for exogenous gene delivery due to their ability to infect both dividing and non-dividing cells, ease of purification to high titers, ability to accommodate long exogenous genes, and their episomal, non-integrating nature [reviewed in (3)]. While later generations of AdV-based vectors have been developed that lack more or all of the AdV genes, the first-generation vectors which are lacking E1 and often also E3 are the most commonly used vector type in the vaccine setting. While the deletion of the E1 region results in replication deficiency and creates space for the transgene-encoding cassette, the deletion of E3 prevents the downregulation of the major histocompatibility complex I on transduced cells (4), resulting in improved immunogenicity and allowing for insertion of larger transgenes.

Originally, the species C HAdV types 2 and 5 were most often used for vector development (5–9), but their applicability had been questioned due to high levels of pre-existing immunity in many populations, especially in Africa and Asia (10, 11), and after high pre-existing immunity against HAdV-C5 was found to be linked to higher HIV-1 infection rates of male, non-circumcised vaccinees in the phase IIb STEP vaccine trial, the research focus shifted to so-called “rare” HAdV types with low seroprevalence for the development of new HAdV-based vectors. In addition to the seroprevalence, another important factor to consider when selecting AdV types for future vector development is their immunogenicity, which refers to the ability to trigger an immune response in the host, including the activation of innate immune mechanisms and of antigen presenting immune cells and the promotion of cellular immunity. AdV types with high immunogenicity can effectively activate the immune system and enhance the immune response to the antigens carried as transgenes by the vector, thus acting as adjuvants and improving the protective efficacy of the vaccine. HAdV types differ in their immunogenicity, resulting in differential activation of antigen-presenting cells and, consequently, of CD8^+^ T cells [reviewed in (12)]. A range of HAdV types other than HAdV-C5 have been evaluated as vectors for immunization, with some of them showing efficacy comparable to that of HAdV-C5 (**Table 1**). While many HAdV have been shown to be clearly inferior to HAdV-C5 with regard to their ability to induce transgene-specific CD8^+^ T cell responses, a few, most notably HAdV-D26, -D28, -C6 and -E4 showed good potential in immunization models. Subsequently, vectors based on HAdV-C5 and -D26 and the chimpanzee adenovirus Y25 based ChAdOx-1 vector have proven highly effective in the last few years in their widespread use for immunization against SARS-CoV-2 (26–29) and on a smaller scale against Ebola virus (30, 31). The very successful deployment of these vaccines has shown the great potential of AdV-based vectors.

**Table 1:** Immunogenicity data of different HAdV type based vectors in pre-clinical models.

HAdV type	vaccine antigen	vector dose [vp]	CD8^+^ T cell detection	CD8^+^ T cell response	fold-change compared to HAdV-C5	Reference
Ad4, Ad5	Zika prME	10^10^	Elispot	Ad5: 80000 SFC/10^6^ Ad4: 55000 SFC/10^6^	**Ad4: 0.69**	(13)
Ad4, Ad5	Zika prME	10^10^	Elispot	Ad5: 3000 SFC/10^6^ Ad4: 1000 SFC/10^6^	Ad4: 0.33	(14)
Ad4, Ad5	Influenza HA	10^10^	Elispot	Ad4: 450 SFC/10^6^ Ad5: 800 SFC/10^6^	**Ad4: 0.56**	(14)
Ad5, Ad6, Ad24, Ad26, Ad35, Ad34	HIV Gag	10^10^	Elispot	Ad5: 900 SFC/10^6^ Ad6: 650 SFC/10^6^ Ad26: 500 SFC/10^6^ Ad24: 150 SFC/10^6^ Ad35: 150 SFC/10^6^ Ad34: 100 SFC/10^6^	**Ad6: 0.72** **Ad26: 0.55** Ad24: 0.17 Ad35: 0.17 Ad34: 0.11	(15)
Ad5, Ad26, Ad35	HIV Gag	10^8^	TetI, day 15	Ad5: 10000 cells/10^6^ Ad26: 3000 cells/10^6^ Ad35: 1000 cells/10^6^	Ad26: 0.33 Ad35: 0.1	(16)
Ad5, Ad26, Ad35, Ad48	LCMV GP	10^10^	TetI, day 7	Ad5: 8.1% Ad26: 2.5% Ad35: 2.2% Ad48: 1.8%	Ad26: 0.31 Ad35: 0.27 Ad48: 0.22	(17)
Ad5, Ad11, Ad35, Ad50, Ad26, Ad48, (Ad49)	SIV Gag	10^9^	Elispot	Ad5: 500 SFC/10^6^ Ad11: 220 SFC/10^6^ Ad35: 200 SFC/10^6^ Ad50: 75 SFC/10^6^ Ad26: 500 SFC/10^6^ Ad48: 250 SFC/10^6^ (Ad49: 125 SFC/10^6^)	Ad11: 0.44 Ad35: 0.4 Ad50: 0.15 **Ad26: 1** Ad48: 0.5 (Ad49: 0.25)	(18)
Ad5, Ad48, Ad50	Ovalbumin	10^9^	Dextramer	Ad5: 4% Ad48: 0.7% Ad50: 0.9%	Ad48: 0.18 Ad50: 0.23	(19)
Ad5, (Ad28), Ad35	SIV Gag	10^8^	Tet, day 21	Ad5: 15% (Ad28: 10%) Ad35: 1%	**(Ad28: 0.66)** Ad35: 0.07	(20)
Ad5, Ad26	HIV Gag	10^10^	Tet, day 21	Ad5: 40000 cells/10^6^ Ad26: 50000 cells/10^6^	**Ad26: 1.25**	(21)
Ad5, Ad35, Ad11	SIV Gag	10^8^	Tet, day 10	Ad5: 4.8% Ad11: 1% Ad35: 1%	Ad11: 0.21 Ad35: 0.21	(22)
Ad5, Ad35	SIV Gag	10^8^	Elispot, day 14	Ad5: 530 SFC/10^6^ Ad35: 250 SFC/10^6^	Ad35: 0.47	(23)
Ad5, Ad14, (Ad28), Ad35	Influenza NP	10^8^	ICS, day 14	Ad5: 1.1% Ad14: 0.5% (Ad28: 0.6%) Ad35: 0.1%	Ad14: 0.45 **(Ad28: 0.55)** Ad35: 0.09	(24)
Ad5, Ad24, Ad34, Ad35	HIV Gag	10^10^	Elispot	Ad5: 800 SFC/10^6^ Ad24: 325 SFC/10^6^ Ad34: 105 SFC/10^6^ Ad35: 153 SFC/10^6^	Ad24: 0.41 Ad34: 0.13 Ad35: 0.19	(25)

The literature was searched for side-by-side comparisons of HAdV-C5-based vectors with vectors based on other HAdV types. The mean levels of CD8^+^ T cell responses to the vaccine antigens were extracted and fold-change for the rare HAdV type based vectors compared to HAdV-C5 based vectors was calculated. HAdV types resulting in more than 50% of the CD8^+^ T cell response induced by HAdV-C5 are written in bold type. HAdV types shown in brackets were not part of this study.

The large-scale deployment also means, however, that a considerable part of the population now has some degree of immunity against these vector types. This vaccine-induced vector immunity may make these vector types less effective if applied again in a boost immunization or in the future as the vector for a different immunization. Thus, it is highly desirable to enlarge the repertoire of effective AdV-based vaccine vectors for future vaccine development and epidemic preparedness. Toward this goal, we have investigated in previous studies the seroprevalence of 39 HAdV types in a cohort of medical students (32) and in a cohort of patients with neuromuscular disease (33) and identified rare HAdV types with significantly lower seroprevalence compared to HAdV-C5. In the study presented here, we aimed to characterize the same broad range of 39 HAdV types with regard to their immunogenicity. Although many reports have constructed AdV-based vectors and evaluated their immunogenicity *in vitro* and *in vivo*, most studies have only focused on a few individual AdV types (**Table 1**), and there is currently a lack of such systematic studies on a wide range of AdV types. In the present study, we screened 40 AdV types from species A through G *in vitro*, including 39 HAdV types and the chimpanzee AdV Y25 derived vector ChAdOx-1, for their impact on the proliferation capacity of CD8^+^ T cells, as well as their influence on bone-marrow-derived dendritic cell (bmDC) activation. Ultimately, we identified the most promising types of HAdVs for future vector development.

## Materials and methods

### Viruses and viral vectors

In this study, a total of 39 types of HAdVs were employed, consisting of 34 wild-type HAdVs (HAdV-A12, -A18, -A31, -B3, -B7, -B11, -B14, -B21, -B34, -B35, -C1, -C2, -C5, -C6, -D8, -D9, -D10, -D13, -D17, -D20, -D24, -D25, -D26, -D27, -D33, -D37, -D70, -D73, -D74, -D75, -D80, -E4, -F41, and -G52) and five adenoviral vectors encoding eGFP and/or firefly luciferase (Ad16.GLN, Ad65.GLN, and Ad69.GLN (encoding eGFP and luciferase), Ad48.GFP and Ad50.Luc). Comprehensive details regarding the cultivation and purification of these HAdVs and HAdV-based vectors can be found in our previous study (32). In addition, the chimpanzee adenovirus-derived vector ChAdOx1-eGFP [kindly provided by Sarah Gilbert, University of Oxford (34)] was also included in this study.

For UV inactivation, AdV particles in 30 µl of PBS were irradiated with UV light (254 nm) for 30 minutes at 4°C.

### Mice

C57BL/6 background human CD46-transgenic mice (B6-CD46tg) were obtained by backcrossing IFNAR^-/-^SLAM Ge CD46 Ge S1 mice (35) onto C57BL/6 background and selecting single transgenic mice, and were kindly provided by Matthias Tenbusch, University of Erlangen-Nürnberg, Gemany, with permission from Roberto Cattaneo, Mayo Clinic, Rochester, MN. T cell receptor-transgenic C57BL/6 mice express a CD8^+^ T cell receptor specific for the immunodominant Friend virus CD8^+^ T cell epitope GagL_85-93_ (36).

All mice were bred and maintained in the animal facility of the Institute for Virology at the University Hospital Essen. All procedures were carried out in compliance with national regulations and followed the institutional guidelines of the University Hospital Essen, Essen, Germany.

### Isolation and culture of bmDCs

Bone marrow-derived dendritic cells (bmDCs) were cultured from the bone marrow of B6-CD46tg mice. The femurs of the mice were flushed to obtain the bone marrow, which was subsequently cultured in R10 medium (RPMI-1640 medium supplemented with 10% heat-inactivated FCS, 50 μg/ml gentamicin, 20 μg/ml ciprofloxacin, 2 mM L-glutamine, 50 μM β-mercaptoethanol, 10 mM HEPES and 1 mM sodium pyruvate) plus 20 ng/ml GM-CSF and 1.25 ng/ml IL-4, with an addition of an equal volume of fresh medium on day 4. The bone marrow cells were maintained at 37°C, 5% CO2 and 95% humidity, and loosely adherent cells were harvested on the seventh day as bmDCs for further experiments (37).

### *In vitro* bmDC stimulation assay

The bmDC stimulation assay was performed in 96-well plates, wherein the bmDCs were transduced with various types of HAdV at a multiplicity of infection (MOI) of 1000. In addition, a negative control devoid of any stimulant and a positive control stimulated with 10 or 100 μg/ml LPS, were included. bmDCs were fixed with 2% PFA at 24 h post-transduction, and the cells were stained with anti-CD11c-BUV496 (clone HL3, BD Biosciences, Heidelberg, Germany), anti-MHC-I-AF647 (clone KH95, BioLegend, Fell, Germany), anti-MHC-II-PE/Dazzle594 (clone M5/115.14.2, BioLegend), anti-CD40-AF488 (clone HM40-3, BioLegend), anti-CD80-PE-Cy5 (clone 16-10A1, BioLegend), anti-CD86-BV510 (clone GL-1, BioLegend), anti-4-1BBL-PE (clone TKS-1, BioLegend), anti-CD252-PE-Cy7 (clone RM134L, BioLegend), anti-CD54-BUV737 (clone 3E2, BD Biosciences), anti-CD83-BV650 (clone Michel-19, BioLegend), anti-PD-L1-PerCP (clone MIH5, BD Biosciences), anti-PD-L2-BV421 (clone TY25, BioLegend) and Fixable Viability Dye eFluor 780 (eBioscience, Frankfurt, Germany). Data were acquired on a BD FACSymphony A5 flow cytometer (BD Biosciences) and analyzed using FlowJo software (TreeStar, Ashland, OR).

### *In vitro* proliferation assay

Friend virus GagL_85-93_-specific CD8^+^ T cells were isolated from spleen cells of the T cell receptor transgenic mice by magnetic cell sorting using CD8 microbeads (Miltenyi, Bergisch-Gladbach, Germany). Subsequently, the isolated T cells were labeled with 1.5 μM carboxyfluorescein succinimidyl ester (CFSE) to facilitate tracking of their proliferation. The bmDCs were loaded with the peptide GagL_85-93_ and simultaneously transduced with HAdVs at a multiplicity of infection (MOI) of 1000 and then co-cultured with CFSE-labelled CD8^+^ T cells at an initial ratio of 1:2.5 for 3 days. CFSE intensity was analysed using flow cytometry after staining CD8^+^ T cells with BV421-anti-CD8 antibody (clone 53-6.7, BioLegend). Untreated bmDCs were used in the negative control coculture, while bmDCs loaded exclusively with the peptide without undergoing transduction by HAdVs were employed in the positive control stimulation to provide a baseline for comparison.

Additional proliferation assays were conducted in a transwell system, where HAdV-transduced bmDCs were placed in the upper transwell chamber, while GagL_85-93_-loaded bmDCs, as well as CD8^+^ T cells, were placed in the lower chamber. Following a three-day co-incubation period, the CD8^+^ T cells were subjected to staining and analysis, as described above.

Data were acquired on a BD FACSymphony A5 flow cytometer (BD Biosciences) and analyzed using FlowJo software (TreeStar, Ashland, OR). The division index of individual samples was calculated in FlowJo software as the total number of cell divisions divided by the total number of cells at the start of the culture of that individual sample as calculated from the division peaks.

### Bead-based multiplex cytokine assay

For cytokine analysis, bmDCs were transduced with different HAdVs or left unstimulated (negative control) or stimulated with 10 or 100 μg/ml LPS (positive controls). The culture supernatants were harvested and subjected to analysis of mouse cytokines using the bead-based LEGENDplex multi-analyte flow assay kit (BioLegend), which encompassed a range of cytokines including interferons (IFN-α, -β, -γ), interleukins (IL-1β, -6, -10, -12), chemokines (CCL2, CCL5, CXCL1), TNF-α, and GM-CSF. Experiments were performed following the manufacturer’s instructions. Data were acquired by flow cytometry on a BD FACSymphony A5 flow cytometer (BD Biosciences) and analyzed by Legendplex V8.0 software (BioLegend).

### Statistical analysis

Data were analyzed for statistically significant differences using GraphPad Prism software version 8, applying a one-way analysis of variance (ANOVA) for the comparison among multiple groups or an unpaired t test for pairwise comparisons. Univariate regression analysis was performed by Pearson correlation analysis in GraphPad Prism software. Multivariate regression analysis was performed by random forest analysis in R software using the packages randomForest and randomForestExplainer. Bayesian regression analysis was performed in R software using the package rstanarm using the mean fold-change of the division index of CD8^+^ T cells in the *in vitro* proliferation assay, the mean fold-change of bmDC surface marker and the mean fold-change of cytokine secretion levels, and the classification of HAdV-C5, -C6, -D26, -E4 and ChAdOx as “favorable vectors” and HAdV-B11, -B14, -B34, -B35, -B50, -D24 and -D48 as “unfavorable vectors” according to previously published data (**Table 1**). The results of the Bayesian regression analysis were visualized using the packages bayesplot, ggplot and ggforce.

## Results

### Reduction in CD8^+^ T cell proliferation after stimulation with AdV-transduced bmDCs

We observed in the past that vectors based on different HAdV types encoding the same transgene had strikingly different potential to induce transgene-specific CD8^+^ T cells *in vivo* and to stimulate the proliferation of transgene-specific CD8^+^ T cells in an *in vitro* proliferation assay (19). To inform future selection of HAdV types for the development of new HAdV-based vectors, we decided to screen a wide range of HAdV types in an *in vitro* proliferation assay and to characterize their influence on antigen-presenting cells.

**Figure 1:**
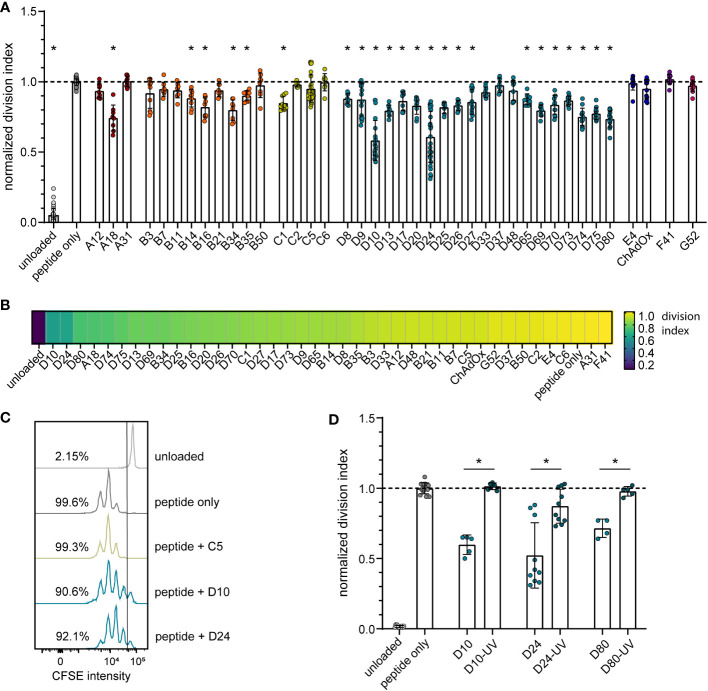
Reduction in CD8^+^ T cell proliferation after transduction of bmDCs with AdV. A CD8^+^ T cell proliferation assay was performed using FV GagL_85-93_ specific CD8^+^ T cells and bmDCs loaded with the GagL_85-93_ peptide with and without additional transduction with the indicated HAdV types or ChAdOx. **(A)** Proliferation of the CD8^+^ T cells was analyzed by dilution of CFSE staining intensity after 3 days of coculture, and the division index of the CD8^+^ T cells was calculated and normalized to the division index of the peptide-only stimulation. **(B)** A heatmap representation of the mean normalized division indices of CD8^+^ T cells stimulated with peptide and the indicated AdV types. **(C)** An overlay of representative histograms of different CD8^+^ T cell stimulations. **(D)** The influence on CD8^+^ T cell proliferation of bmDCs transduced with different untreated and UV-inactivated HAdV; the graph shows the division index normalized to the peptide-only control. Each dot indicates an individual sample, bars indicate mean values, and whiskers indicate the standard deviation. Data were acquired in at least two independent experiments **(A, B, D)**. The dashed line indicates the normalized value of 1 **(A, D)**, * indicates a statistically significant difference compared to the peptide-only control (**A**; *P* < 0.05, one-way ANOVA) or between the indicated groups (**D**; *P* < 0.05, unpaired t test).

To analyze the impact of different HAdV types on the proliferation capacity of transgene-specific CD8^+^ T cells, we used murine TCR transgenic CD8^+^ T cells that are specific for the Leader-Gag-derived epitope GagL_85-93_ of the Friend retrovirus, which is a model retrovirus infection that has been used by us and others in the past to study immune mechanisms and vaccine approaches targeting retrovirus infections [reviewed in (38)]. While the CD8^+^ T cells proliferated strongly in the *in vitro* proliferation assay when they were coincubated with bmDCs loaded with the GagL_85-93_ peptide alone, their proliferation activity varied greatly when the peptide-loaded bmDCs were transduced with different HAdV types (**Figure 1A–C**). While some HAdV types, such as HAdV-A31, -B50, -C2, -C5, -C6, -D37, -E4, -F41 and ‐G52, did not affect CD8^+^ T cell proliferation, many other HAdV types, notably almost all tested species D types but also HAdV-A18, some species B types and, to a moderate extent, HAdV-C1, led to significantly reduced CD8^+^ T cell proliferation. When we performed a complementary experiment with the most strongly inhibitory HAdV types, HAdV-D10, -D24 and -D80, we found that the inhibitory effect was abolished when the viruses were UV-inactivated (**Figure 1D**).

### Influence of AdV transduction on bmDC surface marker expression

**Figure 2:**
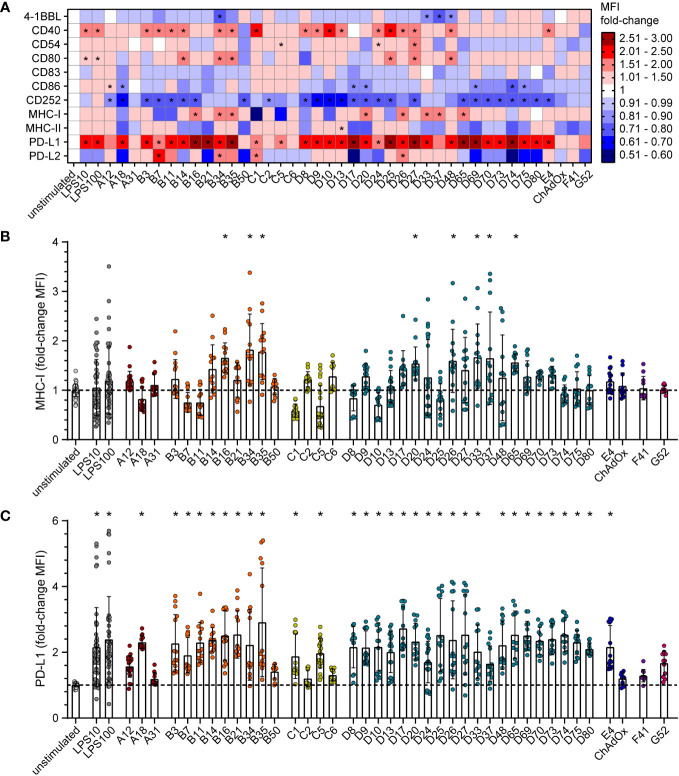
Differential expression of bmDC surface markers after transduction with AdV. bmDCs were transduced with the indicated AdV types or stimulated with 10 µg/ml or 100 µg/ml LPS. After 24 hours, the expression of surface molecules was analyzed by flow cytometry. The median fluorescence intensity (MFI) of the surface markers was determined, and fold-changes compared to the unstimulated bmDCs were calculated. **(A)** A heatmap representation of the mean fold-change of the MFI of the indicated surface markers after stimulation with the indicated AdV types. **(B, C)** Dot plot representations of the MFI fold-change for MHC-I **(B)** and PD-L1 **(C)**. Each dot indicates an individual sample **(B, C)**, bars indicate mean values, and whiskers indicate the standard deviation. Data were acquired in at least two independent experiments **(A–C)**. The dashed line indicates the normalized value of 1 **(B, C)**, * indicates a statistically significant difference compared to the unstimulated control (*P* < 0.05, one-way ANOVA).

**Figure 3:**
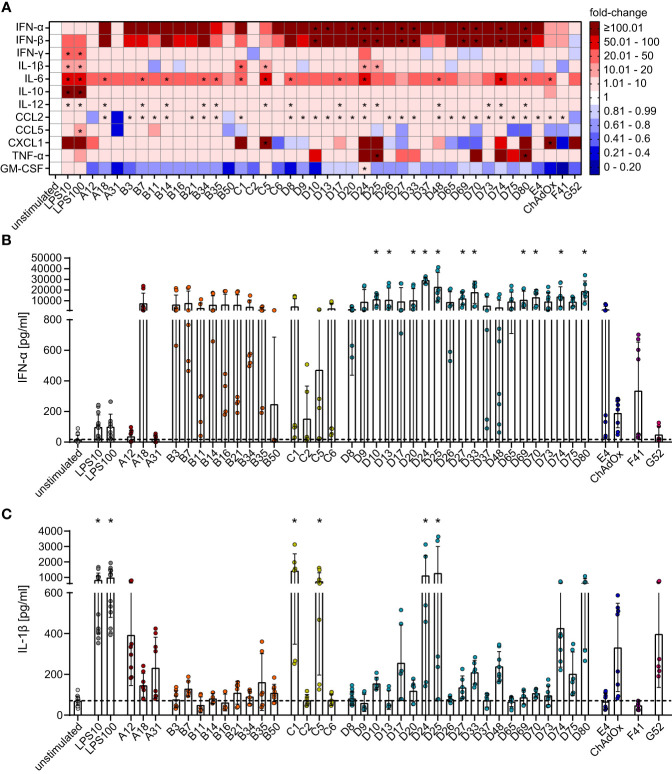
Differential secretion of cytokines by bmDCs after transduction with AdV. bmDCs were transduced with the indicated AdV types or stimulated with 10 µg/ml or 100 µg/ml LPS. After 24 hours, the supernatants were collected and analyzed for the presence of the indicated cytokines. The cytokine concentrations were determined and fold-changes compared to the unstimulated bmDCs were calculated. **(A)** A heatmap representation of the mean fold-change of the concentrations of the indicated cytokines after stimulation with the indicated AdV types. **(B, C)** Dot plot representations of the concentrations of IFN-α **(B)** and IL-1β **(C)** in the supernatant of bmDCs after the indicated stimulations. Each dot indicates an individual sample **(B, C)**, bars indicate mean values, and whiskers indicate the standard deviation. Data were acquired in at least two independent experiments **(A–C)**. The dashed line indicates the mean concentration of the indicated cytokine in the supernatant of unstimulated bmDCs **(B, C)**, * indicates a statistically significant difference compared to the unstimulated control (*P* < 0.05, one-way ANOVA).

To gain mechanistic insight into the differential effect of HAdV types on CD8^+^ T cell proliferation, we characterized the impact of HAdV transduction of bmDCs on the expression of stimulatory and inhibitory DC surface markers (**Figure 2**; **Supplementary Figure S1**) and on the secretion of cytokines by the bmDCs (**Figure 3**; **Supplementary Figure S2**). The bmDC surface molecules showed different patterns in response to transduction by different HAdV types. The surface expression of the co-stimulatory molecules 4‐1BBL, CD83 and CD86 was only slightly affected by most of the tested HAdV types and only significantly reduced by a few species B and D HAdV types (4-1BBL: HAdV-B34, ‐D33, ‐D37, -D48; CD86: HAdV-A18, -D17, -D20, -D69, -D74, -D75; **Figure 2A**). The expression of the co-stimulatory molecule CD40 was increased by a majority of the tested HAdV types, whereas the expression of CD54 and CD80 was increased to a lesser extent by some HAdV types and decreased by others, and the expression of CD252, on the other hand, was decreased by all tested HAdV types. The expression of the peptide-presenting molecules MHC-I (**Figures 2A, B**) and MHC-II was differentially and in many cases opposingly influenced by the different HAdV types, with a significant upregulation of MHC-I observed only for HAdV-B16, -B34, -B35, -D20, -D26, -D33, -D37 and -D65. The expression of the inhibitory molecule PD-L1 (**Figure 2C**) was significantly upregulated by most of the tested HAdV types, with the strongest induction (> 2.5-fold) observed for HAdV-B16, -B21, -B35, -D17, -D25, -D27, -D65, -D69 and -D74. Expression of the inhibitory molecule PD-L2, on the other hand, was not as strongly affected and was significantly upregulated only by HAdV-B7, -B34, -C1, and -D26, whereas the expression showed a strong trend toward a decrease after transduction with other HAdV types such as HAdV-A18, -B21, -B50, -D17 and -D74. Notably, the single animal-derived AdV type, ChAdOx, had limited impact on any of the markers, similar to HAdV types A31, C6, F41, and G52. In addition, comparisons at the species level showed that HAdVs of species B and D had a greater impact on the expression of bmDC surface molecules, while species F and G HAdVs exerted a relatively mediocre effect.

### Influence of AdV transduction on bmDC cytokine secretion

The effect of bmDC HAdV transduction on cytokine production revealed a more homogenous picture for most of the analytes (**Figure 3A**). Compared to the unstimulated control, HAdV transduction resulted in a substantial increase in the concentrations of most cytokines, with the most pronounced effects observed for IFN-α (**Figures 3A, B**) and IFN-β (**Figure 3A**), which showed a clear trend toward strong induction by all HAdV types and statistically significant increases by most species D HAdV types. Concentrations of IL-6, IL-12, and CCL2 were also significantly upregulated, but to a lesser extent than IFN-α and IFN-β, and did not differ distinctly across HAdV species. On the other hand, the secretion of IL-1β (**Figures 3A, C**), CXCL-1, and TNF-α (**Figure 3A**) was not strongly affected by most HAdVs, and only a few specific HAdV types induced significant increases in their secretion (IL-1β: HAdV-C1, -C5, -D24 and -D25; CXCL1: HAdV-C5 and ChAdOx; TNF-α: HAdV-D25 and -D80), whereas there was a trend toward reduced secretion after transduction with other HAdV types. It is worth noting that the secretion of several cytokines by bmDCs, including IFN-γ, IL-10, and CCL5, was not significantly altered upon transduction with any type of HAdV tested in this study. Interestingly, the secretion of GM-CSF tended to be reduced under the influence of all tested AdV types except for HAdV-C5 and ‐D24.

### Correlation of CD8^+^ T cell proliferation with bmDC surface marker expression and cytokine secretion

**Figure 4:**
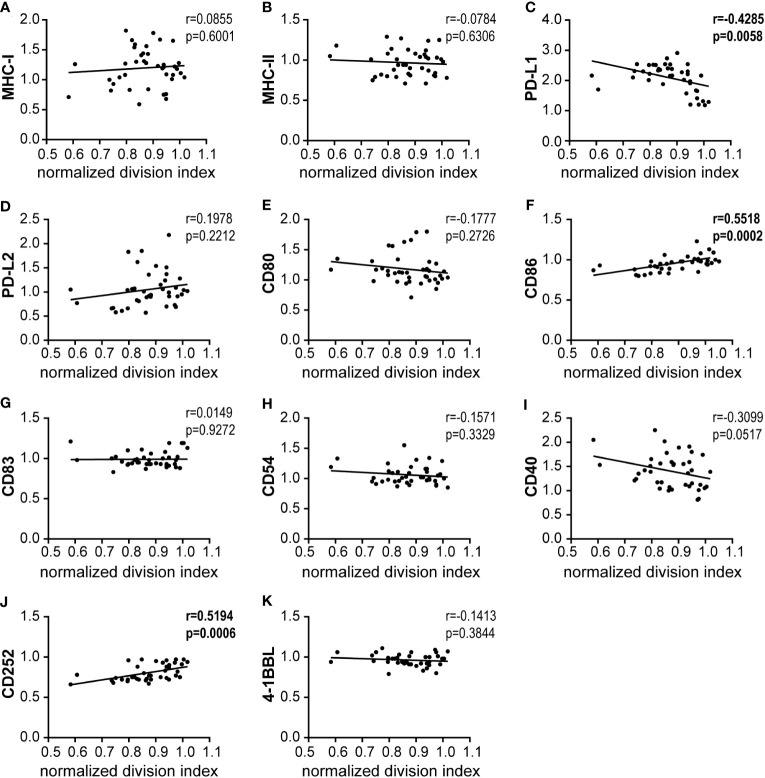
Correlation analysis of bmDC surface markers and CD8^+^ T cell proliferation. **(A–K)** Pairwise Pearson correlation analyses were performed using the mean CD8^+^ T cell division indices as shown in **Figure 1** and the mean fold-change of MFI values for the indicated bmDC surface molecules as shown in **Figure 2**. r, Pearson correlation coefficient; statistically significant correlations are indicated by bold type (*P* < 0.05).

**Figure 5:**
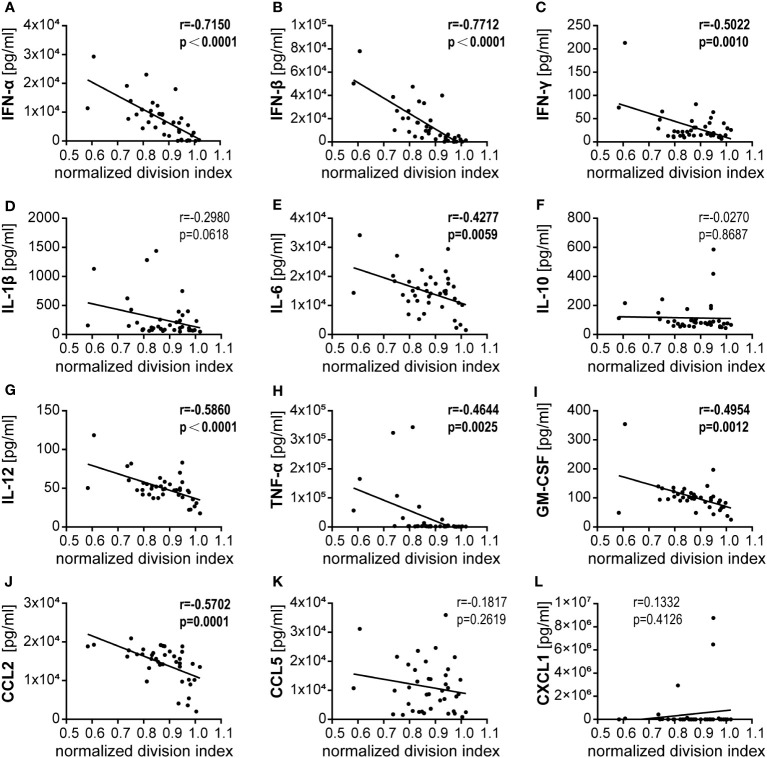
Correlation analysis of bmDC cytokine secretion and CD8^+^ T cell proliferation. **(A–L)** Pairwise Pearson correlation analyses were performed using the mean CD8^+^ T cell division indices as shown in **Figure 1** and the mean concentrations of cytokines secreted by bmDCs as shown in **Figure 3**. r, Pearson correlation coefficient; statistically significant correlations are indicated by bold type (*P* < 0.05).

**Figure 6:**
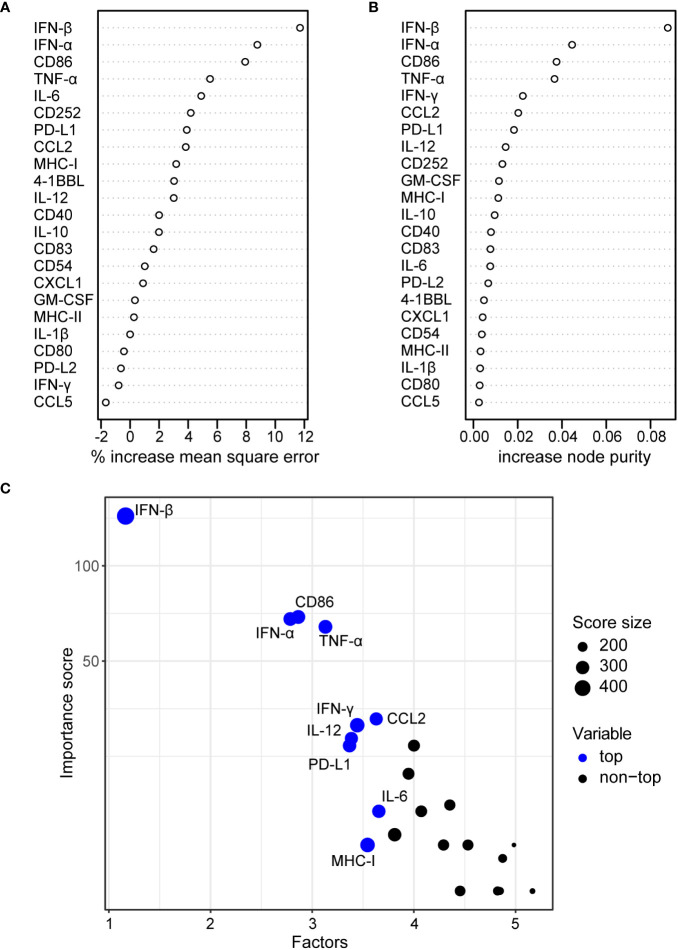
Random forest regression analysis of bmDC cytokine secretion and surface marker expression and CD8^+^ T cell proliferation. A random forest analysis was performed using the mean values for the CD8^+^ T cell division index as shown in **Figure 1**, and the bmDC surface marker expression measured by their MFI as shown in **Figure 2** and the bmDC cytokine secretion as shown in **Figure 3** as explanatory factors. Percent increase in mean square error **(A)** and increase in node purity **(B)** for the 23 tested explanatory factors are shown. The importance plot in **(C)** highlights the variables with the highest importance scores.

In a regression analysis, we analyzed the correlation of the impact of the different HAdV types on bmDC surface marker expression and cytokine secretion levels with the impact on CD8^+^ T cell proliferation capacity. In the analysis of the bmDC surface markers (**Figure 4**), we found a significant and strong positive correlation of CD8^+^ T cell proliferation with the expression levels of the co-stimulatory molecules CD86 (**Figure 4F**) and CD252 (**Figure 4J**) and a negative correlation with the expression level of the inhibitory molecule PD-L1 (**Figure 4C**). We also found significant negative correlations of CD8^+^ T cell proliferation with the secretion of INF-β, IFN-α, IL-12, CCL2, IFN-γ, GM-CSF, TNF-α and IL-6 (in descending order, **Figure 5**). To further investigate the contribution of the individual parameters, we performed a multivariate regression analysis by performing a random forest analysis. This analysis confirmed the results of the univariate analyses and predicted the level of IFN-β as the most important variable for the outcome of the CD8^+^ T cell proliferation rate, followed by IFN-α, CD86 and TNF-α (**Figure 6**).

### Inhibition of CD8^+^ T cell proliferation is mediated by high levels of type I interferons

**Figure 7:**
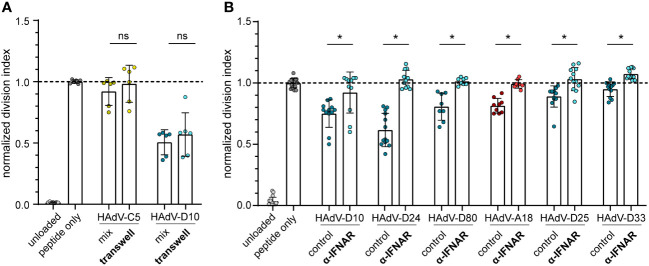
CD8^+^ T cell proliferation in transwell plates and in the presence of type I IFN receptor blocking antibodies. **(A)** A CD8^+^ T cell proliferation assay was performed using FV GagL_85-93_ specific CD8^+^ T cells and bmDCs loaded with GagL_85-93_ peptide, with HAdV-transduced bmDCs either in the same well (mix) or separated from the CD8^+^ T cells and peptide-loaded bmDCs in a transwell chamber (transwell). Proliferation of the CD8^+^ T cells was analyzed by dilution of CFSE staining intensity after 3 days of coculture, and the division index of the CD8^+^ T cells was calculated and normalized to the division index of the peptide-only stimulation. **(B)** A CD8^+^ T cell proliferation assay was performed using FV GagL_85-93_ specific CD8^+^ T cells and bmDCs loaded with GagL_85-93_ peptide and transduced with the indicated HAdV in the absence (control) or presence of an α-IFNAR antibody (α-IFNAR). Proliferation of the CD8^+^ T cells was analyzed by dilution of CFSE staining intensity after 3 days of coculture, and the division index of the CD8^+^ T cells was calculated and normalized to the division index of the peptide-only stimulation. Each dot indicates an individual sample, bars indicate mean values, and whiskers indicate the standard deviation. Data were acquired in at least two independent experiments. The dashed line indicates the normalized value of 1, * indicates a statistically significant difference between the indicated groups (*P* < 0.05, unpaired t test), ns, not significant.

To address these findings experimentally, we performed a CD8^+^ T cell proliferation assay in a transwell system, where the HAdV-transduced bmDCs were spatially separated from the peptide-loaded bmDCs and the CD8^+^ T cells. Here, we found that the inhibition of CD8^+^ T cell proliferation that we observed before when bmDCs were transduced with HAdV-D10 was not changed in the transwell system (**Figure 7A**), confirming that soluble factors mediated the suppressive effect. As the regression analyses ranked IFN-β and IFN-α most highly, we next performed a CD8^+^ T cell proliferation assay in the presence of an interferon α/β receptor (IFNAR) blocking antibody (**Figure 7B**). We selected six HAdV types for which we had observed strong induction of type I interferons (**Figure 3**), four of which had shown the strongest suppression of CD8^+^ T cell proliferation (**Figure 1**). In the control cultures without the addition of IFNAR-blocking antibody, we observed robust suppression of CD8^+^ T cell proliferation as before, which was completely abrogated in the presence of the IFNAR-blocking antibody, confirming the mechanistic role of the induction of type I interferon signaling in the suppression of CD8^+^ T cell proliferation by HAdV in this setting.

### Prediction of favorable HAdV types for vector development

**Figure 8:**
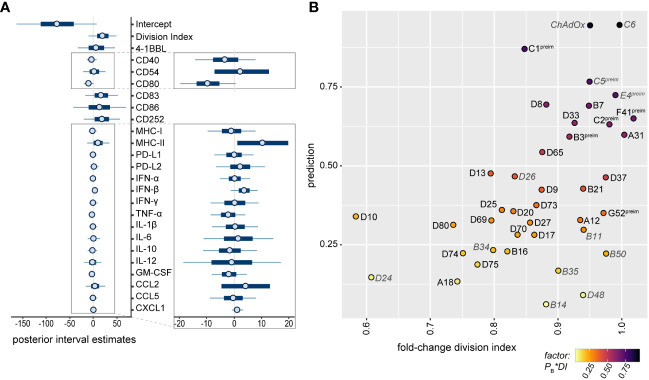
Bayesian regression analysis-based prediction model for the selection of HAdV types for vector development. A Bayesian regression analysis was performed using the mean fold-change of the division index of CD8^+^ T cells in the *in vitro* proliferation assay, the mean fold-change of all tested bmDC surface markers and the mean fold-change of the cytokine secretion levels as factors, and the classification of HAdV-C5, -C6, -D26, -E4 and ChAdOx as “favorable vectors” and HAdV-B11, -B14, -B34, -B35, -B50, -D24 and -D48 as “unfavorable vectors” according to previously published data (**Table 1**) to train the model. Posterior interval estimates are shown in **(A)**, and the right-hand figure shows a zoomed-in graph for improved readability. **(B)** Prediction values for the individual AdV types were plotted against the fold-change division index, and the color of the dots indicates the factor of the Bayes prediction value (P_B_) and the fold-change division index (DI). AdV types that were used to train the model are labelled in grey italics. preim: high pre-existing immunity has been shown previously (32, 33).

For the selection of strong candidates for future HAdV-based vector development, it was of great interest to combine all observations into a statistical model to estimate the potential of the tested HAdV types. We therefore performed a Bayes regression analysis using the data of HAdV-B11, -B14, -B34, -B35, -B50, -C5, -C6, -D24, -D26, -D48 and-E4, which have already been evaluated side-by-side with HAdV-C5 for *in vivo* vaccination efficacy, and of ChAdOx, which has shown its high efficacy as a SARS-CoV-2 vaccine vector, for the calculation of the posteriors. We classified HAdV-C5, -C6, -D26, -E4 and ChAdOx as “favorable vectors” and HAdV-B11, -B14, -B34, -B35, -B50, -D24 and -D48 as “unfavorable vectors” based on the previously published efficacy data, as summarized in **Table 1**. It is noteworthy that in this prediction of good vector candidates, the individual factors are weighted quite differently than in the random forest analysis of the contribution to the repression of CD8^+^ T cell proliferation, and the highest posterior estimates were obtained for the division index of CD8^+^ T cells in the *in vitro* proliferation assay and the expression levels of the DC surface markers MHC-II, CD83, CD86 and CD252, but the secretion levels of some cytokines also tended to have positive posterior estimates (**Figure 8A**). Applying the obtained model to predict promising vector candidates, the HAdV types HAdV-C1, -D8, -B7, -F41, -D33, -C2, -A31, -B3 and -D65 were assigned prediction values higher than 0.5 (in descending order; **Figure 8B**).

## Discussion

AdV-based vectors have been explored during the last decades as gene delivery vectors for gene therapy, oncolytic therapy and immunization, and the last few years have shown the great potential of AdV-based immunization, since AdV-based vaccines were important assets in the effort to control the SARS-CoV-2 pandemic and to curb Ebola virus outbreaks in recent years (39). However, the vast diversity of different HAdV types has not yet been explored for vector development, and systematic comparisons of the immunogenicity of a large number of viruses are currently lacking. In this detailed *in vitro* analysis, we analyzed the effect of 40 AdV types on DC activation and cytokine secretion and on CD8^+^ T cell proliferation capacity and identified HAdV-C1, -D8, -B7, -F41, -D33, -C2, -A31, -B3 and -D65 as having the most favorable immunogenicity profile for vaccine vector development.

In our *in vitro* analysis, we found that the capacity to induce type I interferons correlated with the potential to suppress the proliferative capacity of CD8^+^ T cells. It is recognized that AdV-based vectors are able to stimulate multiple innate immune pathways, whereby they can have an adjuvant effect on the immune response towards the transgene. Multiple innate immune factors influencing the immunogenicity of HAdV types have been described including Toll-like receptors and cGAS/STING [for comprehensive reviews, see (40) and (41)]. Differences in the activation of innate immune mechanisms have been attributed to several factors: in a first step, receptor and integrin binding result in activation of proteins involved in intracellular signaling pathways such as mitogen activated protein kinase by binding to CAR and phosphoinositide 3-kinase by integrin binding, leading to production of pro-inflammatory cytokines such as IL-1α, IL-6, IL-8 and TNF-α (42–45). Differences in receptor usage will result in differential activation of intracellular signaling [reviewed in (46)]. After cell entry, AdV particles have to escape the endosome, which the different AdV types do at different stages along the endosomal-lysosomal axis, resulting in membrane damage and differential release or exposure of endosomal components that induce activation of the NLRP3 inflammasome and of authophagy (47, 48). Finally, differences in the DNA sequence will influence the detection of viral DNA by TLR9 and cGAS/STING and the activation of the subsequent signaling, which results in activation of type I interferon expression (49–51).

Interestingly, it has been shown before that while a moderate ability to stimulate the innate immune system is advantageous for vector potency, excessive stimulation of type I interferons by chimpanzee AdV-based vectors in comparison to HAdV-C5-based vectors resulted in reduced transgene-specific antibody responses (52) and CD8^+^ T cell responses (53), which seemed to correlate with reduced transgene expression in the presence of type I interferons. Furthermore, it was shown that vectors based on HAdV-D28 and HAdV-B35 induced stronger IFN-α *in vitro* and *in vivo*, which resulted in a reduction in transgene expression, a reduced induction of transgene-specific CD8^+^ T cells but also a qualitatively changed CD8^+^ T cell response, with a more pronounced polyfunctional and memory phenotype (54), which the authors attributed to both autocrine and paracrine effects of the elevated IFN-α levels. Taken together, these findings support our *in vitro* data and suggest that the levels of type I interferons play an equally important role in the *in vivo* setting.

Perreau et al. suggested that the number of TLR9 agonist motifs in the HAdV genome correlates with immunogenicity, as they observed a positive correlation between TLR9 agonist motif genome content and the induction of CD86 expression and TNF-α secretion by DCs transduced with HAdV immune complexes (55). Our data also show a positive correlation of the ratio of stimulatory and inhibitory TLR9 motif genome content and the expression level of the DC surface marker CD86 and a negative correlation of the TLR9 motif ratio with the expression level of PD-L1 and the secretion of cytokines, which was most pronounced for type I interferons, CCL2, GM-CSF and IL-12 (**Supplementary Figure 3**). It can still be observed that HAdV types with very similar, low ratios of stimulatory and inhibitory TLR9 motifs induce quite different levels of cytokines, which is in agreement with the finding that multiple innate sensing pathways are involved in HAdV sensing and the induced innate immune response (40), as discussed above.

The model that we developed for the prediction of favorable vector candidates attributes the highest importance to the division index of CD8^+^ T cells in the *in vitro* proliferation assay and the expression levels of the DC surface markers MHC-II, CD83, CD86 and CD252, as well as the secretion levels of some cytokines. Data from *in vivo* studies where different HAdV-based vectors were compared side-by-side demonstrated widely varying potency, and limited induction of cellular immunity was observed for the HAdV types HAdV-B11, -B14, -B34, -B35, -B50, -D24 and -D48 [(15–20, 22–25); **Table 1**]. Interestingly, not all of them showed a strong impact on the CD8^+^ T cell proliferation capacity in our *in vitro* assay, highlighting the complex immune mechanisms underlying the induction of effective immune responses and the value of analyzing a wide range of immune parameters for the prediction of favorable HAdV types for vector development.

According to our Bayes statistic model, the HAdV types HAdV-C1, -D8, -B7, -F41, -D33, -C2, -A31, -B3 and -D65 are the most promising vector candidates. It has to be taken into account, however, that some of these HAdV types showed rather high seroprevalence in our previous studies: neutralizing antibody levels against HAdV-B3, -C1, -C2, and -F41 were similar to those against HAdV-C5, or even higher, in both cohorts (32, 33), precluding their widespread use.

Because of the widespread application of AdV-based vectors for SARS-CoV-2 immunization, it has been noted that in very rare cases, immunization would result in the severe adverse event of vaccine-induced thrombosis with thrombocytopenia syndrome (56, 57), which has been attributed to the induction of autoimmune antibodies directed against platelet factor 4 (PF4), leading to the activation of platelets and granulocytes (58–60). Complex formation of ChAdOx, HAdV-C5 and HAdV-D26 particles with PF4 could be demonstrated experimentally and was found to be in the nanomolar affinity range (61). Importantly, it was recently shown that different HAdV types have distinct affinities for PF4 binding, and HAdV-B11, -B34, -D13 and -D25 exhibited no or very low detectable PF4 binding (62). While the prediction based on our Bayesian statistic modelling is slightly below 0.5 for HAdV-D13, its favorable PF4-non-binding profile may make this an HAdV vector candidate not to be discarded quite yet.

One limitation of our study is that all experiments were performed using murine cells, but there are studies in which murine and human DCs were used side-by-side for AdV infection experiments that support the conclusion that similar results would be obtained using human cells: Johnson et al. performed infection experiments with murine bmDCs and human plasmacytoid DCs and observed the same differences in IFN-α induction by HAdV-C5, -D28 and -B35 in both cell types (54). In a study using only HAdV-C5 based vectors, Strack et al. also showed comparable effects of HAdV-C5 on murine bmDCs and human peripheral blood derived DCs (63). Quinn et al. have performed extensive analyses of innate immune responses using both human peripheral blood mononuclear cells (PBMCs) infected *in vitro* as well as mouse cells from *in vivo* vaccination, and compared the immune response against HAdV-C5 to that against, among others, HAdV-B35, and -D28 (53). They showed that while there was some discordance in up- and down-regulated genes in murine lymph nodes and human PBMCs, expression patterns in murine and human PBMCs were highly comparable. Importantly, they also showed in the *in vivo* model that immunization with HAdV-B35 and -D28 based vectors led to a significantly stronger expression of IFN-α in draining lymph nodes and higher IFN-α levels in the serum compared to HAdV-C5, which also confirms our *in vitro* data. Teigler et al. evaluated the innate immune responses against HAdV-C5, -B35, -D26 and -D48 in rhesus monkeys and in human PBMCs, and reported a strong induction of IFN-α2 in human PBMCs for HAdV‐B35, ‐D26 and -D48 and much less for HAdV-C5 (64), which is in accordance with our findings in murine DCs. Interestingly, Tran et al. have shown in *in vitro* experiments that HAdV-C5 infected human DCs and bystander DCs produced significantly higher levels of a range of cytokines if the HAdV-C5 was complexed with IgG (65), suggesting that cytokine responses *in vivo* may be higher in the presence of pre-existing immunity. Chen et al. analyzed cytokine levels in sera of HAdV-B3 and -B7 infected hospitalized children, and made similar observations of increased levels of CCL-2, IL-6, IL-10, IFN-α and -γ and TNF- α in the infected children in comparison to healthy controls (66). While the authors observed less pronounced IFN-α levels than could be expected from the *in vitro* studies, this may be due to the time point of serum collection. In accordance with the above-mentioned similarities, it has been noted in various reviews (67, 68) that human and mouse DCs share many similarities and that they behave very similarly if they have been prepared, cultured and activated in the same way. Finally, it should be noted that those AdV types that had been most effective in various mouse models have proven their high efficacy in recent years when they were applied as SARS-CoV-2 vaccines, lending validity to results obtained in mouse experiments. Overall, we conclude that the results obtained in our *in vitro* assays with murine cells are meaningful and valid also for possible translation into human use in the future, and that vectors derived from the identified favorable HAdV types can be expected to also have favorable *in vivo* immunogenicity.

Our data provide important insight into the different immunogenicity profiles of 40 AdV types. Taken together with our previously reported data on seroprevalence, we suggest that HAdV-D8, -B7, -D33, -A31 and -D65 are the most favorable HAdV types to be explored as vaccine vectors and evaluated *in vivo* in animal models in the future.

## Data availability statement

The original contributions presented in the study are included in the article/**Supplementary Material**, further inquiries can be directed to the corresponding author/s.

## Ethics statement

The animal study was approved by Internal Review Board of the Animal Facility of the University Hospital Essen. The study was conducted in accordance with the local legislation and institutional requirements.

## Author contributions

XW: Data curation, Formal Analysis, Funding acquisition, Investigation, Visualization, Writing – original draft, Writing – review & editing. MH: Data curation, Formal Analysis, Investigation, Methodology, Writing – review & editing. WZ: Investigation, Methodology, Resources, Writing – review & editing. AE: Conceptualization, Investigation, Resources, Writing – review & editing. WB: Conceptualization, Data curation, Formal Analysis, Funding acquisition, Methodology, Project administration, Resources, Supervision, Validation, Visualization, Writing – original draft, Writing – review & editing.
