# Supplementary material for: Comparative analysis of the impact of 40 adenovirus types on dendritic cell activation and CD8+ T cell proliferation capacity for the identification of favorable immunization vector candidates
Source: Front Immunol. 2023 Oct 17;14:1286622. doi: 10.3389/fimmu.2023.1286622 (PMC10616870; doi:10.3389/fimmu.2023.1286622)
Supplement: Supplementary file 1 [file DataSheet_1.pdf]

Supplementary Figure S1

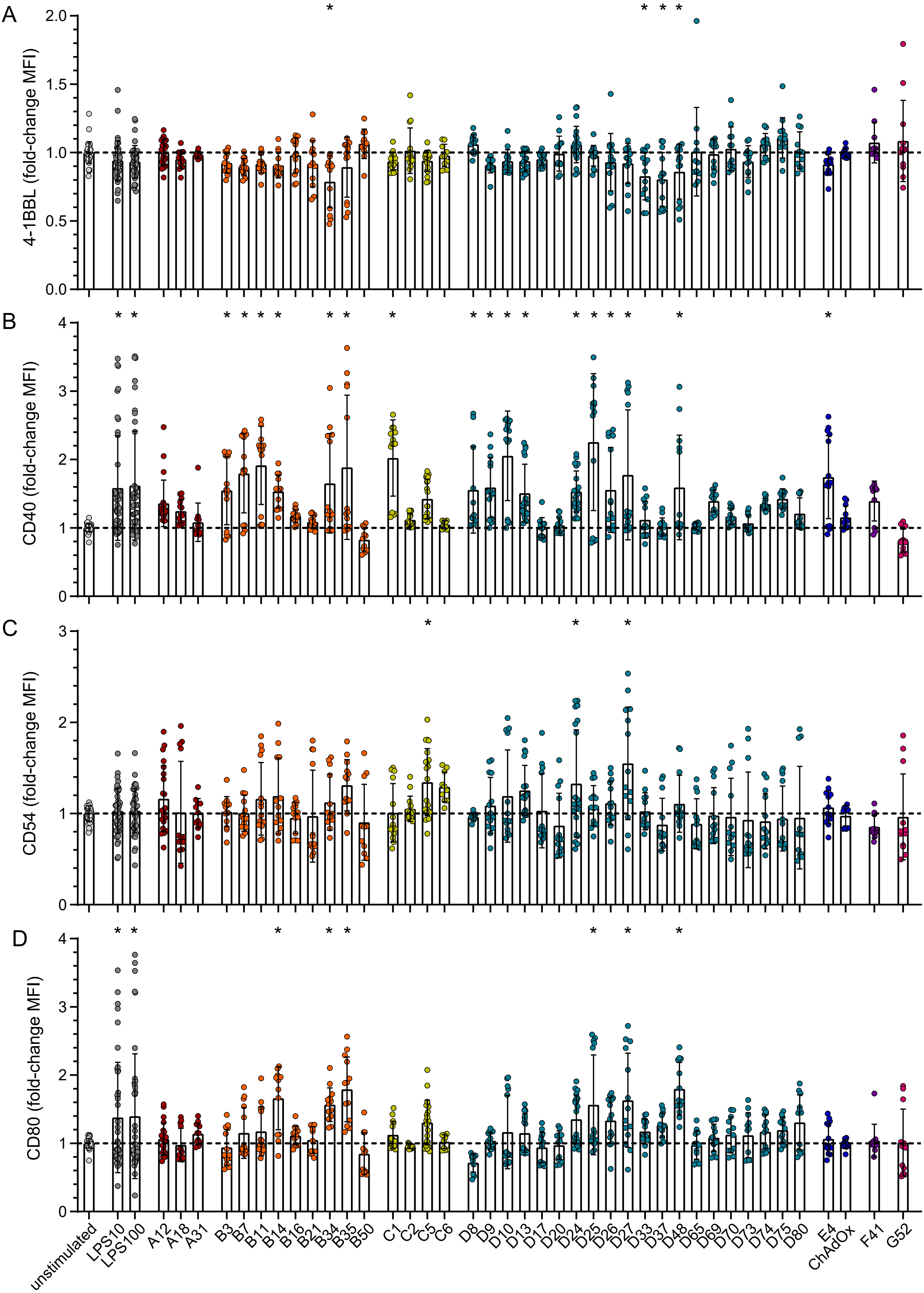

Supplementary Figure S1 (continued)

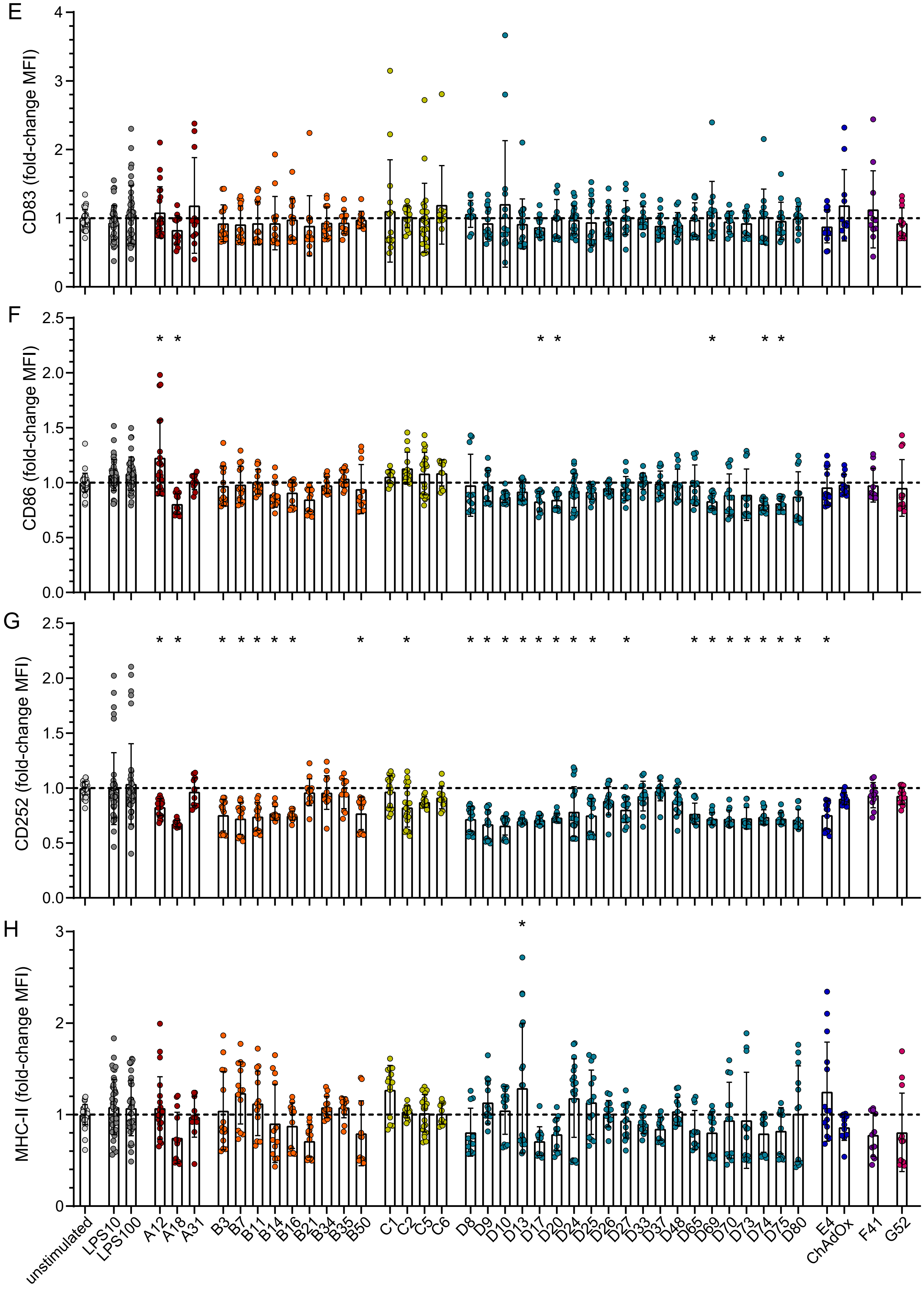

Supplementary Figure S1 (continued)

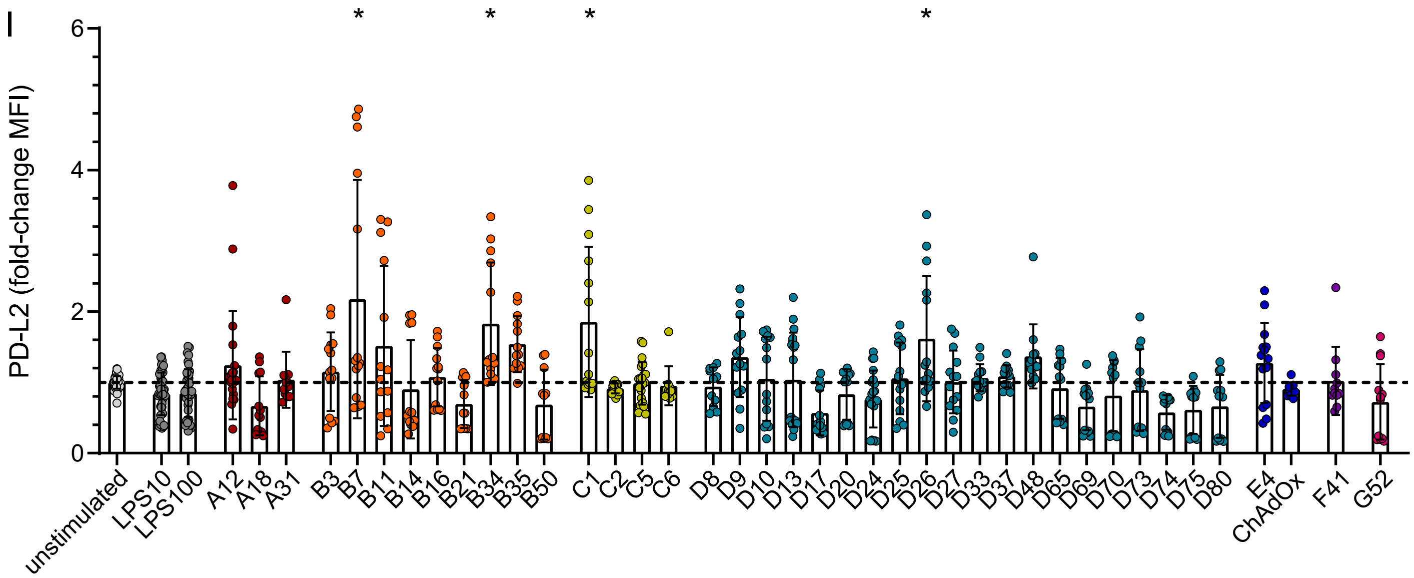


**Differential expression of bmDC surface markers after transduction with AdV.** bmDC were transduced with the indicated AdV types or stimulated with 10 µg/ml or 100 µg/ml LPS. After 24 hours, the expression of surface molecules was analyzed by flow cytometry. The median fluorescence intensity (MFI) of the surface markers was determined and fold-changes compared to the unstimulated bmDCs were calculated. (A-I) Dot plot representations of the MFI fold-change for the indicated surface markers.

Each dot indicates an individual sample, bars indicate mean values, whiskers indicate the standard deviation. Data were acquired in at least two independent experiments. The dashed line indicates the normalized value of 1, * indicates a statistically significant difference compared to the unstimulated control ($P < 0.05$, One-Way ANOVA).

Supplementary Figure S2

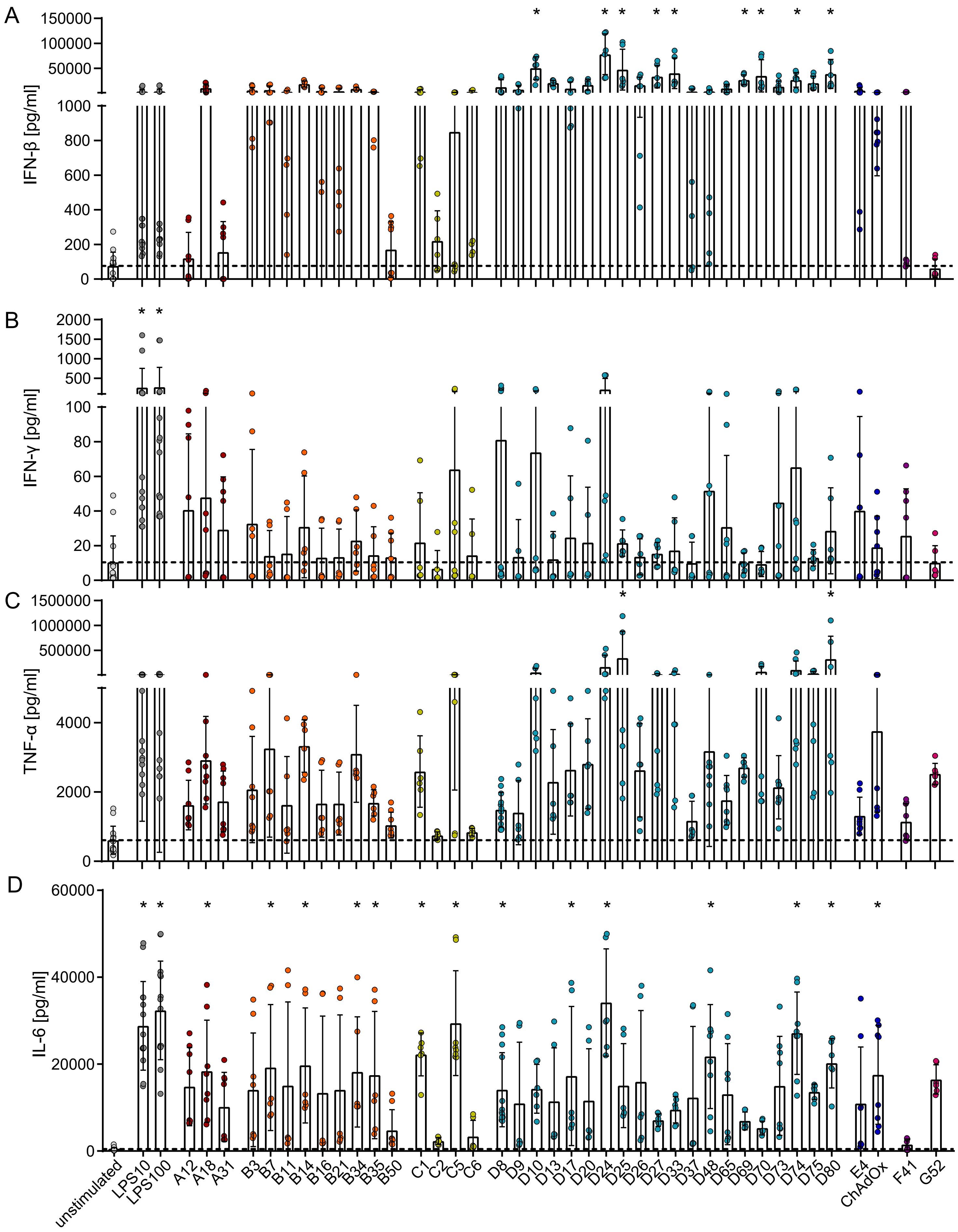

Supplementary Figure S2 (continued)

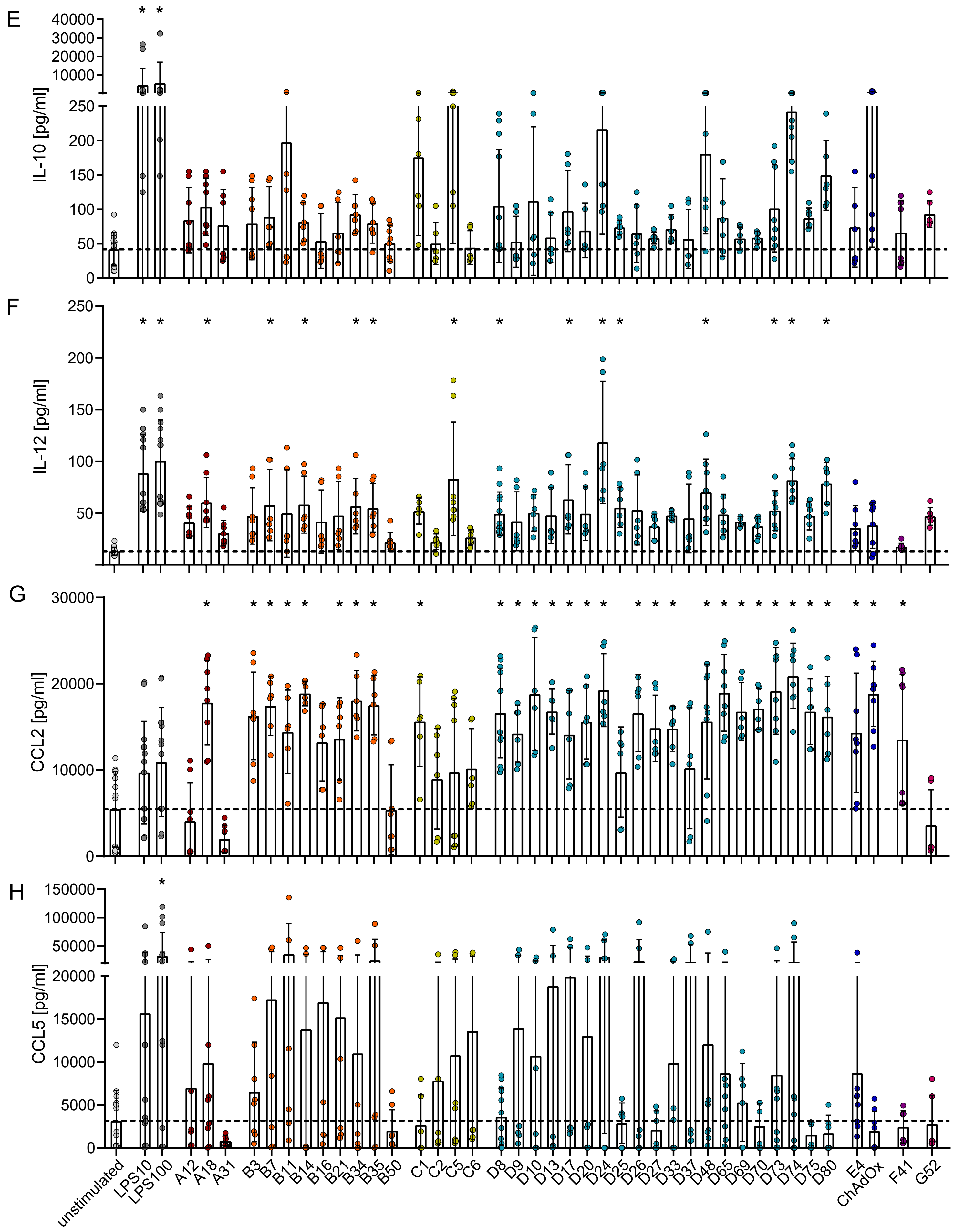

Supplementary Figure S2 (continued)

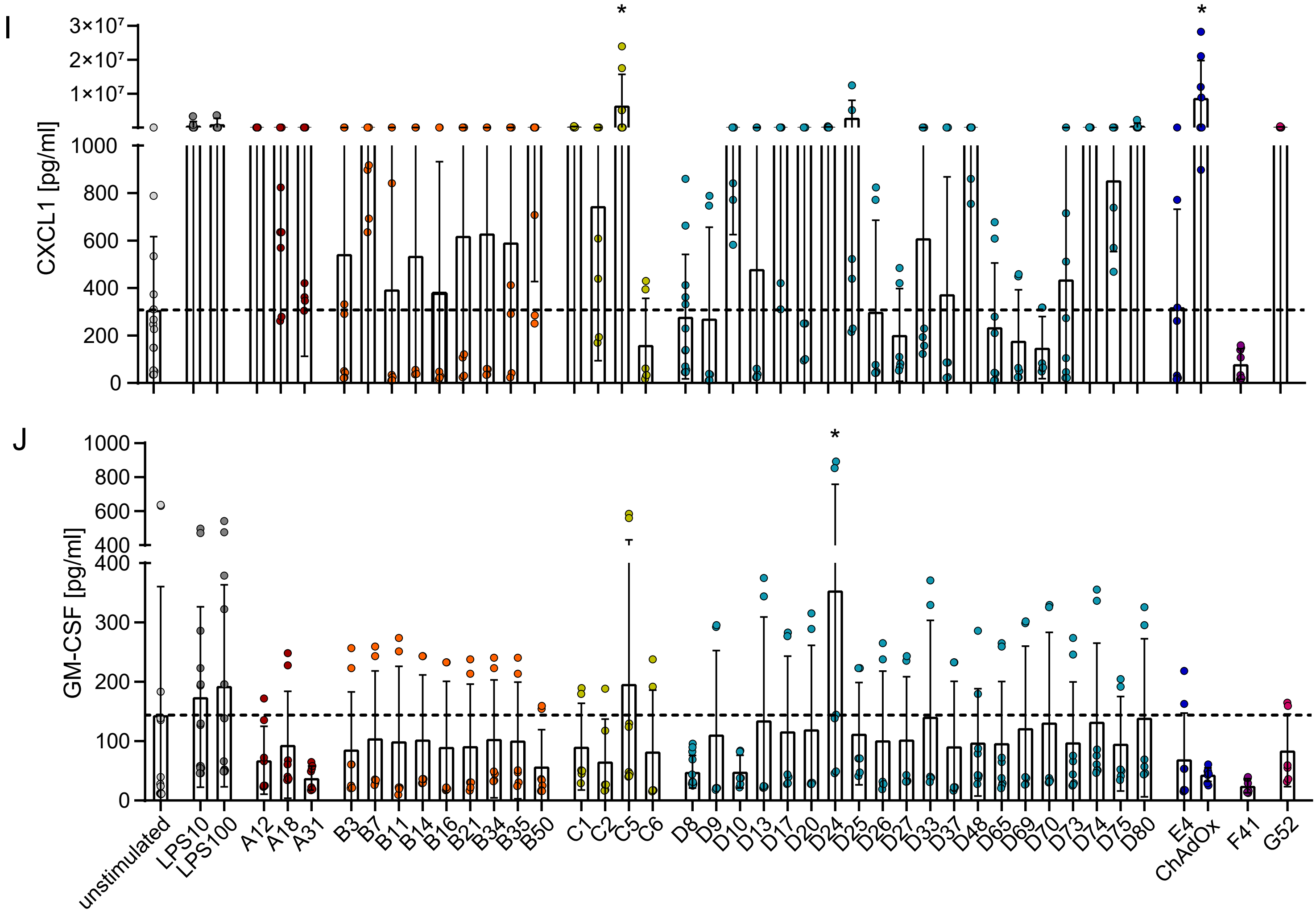


**Differential secretion of cytokines by bmDC after transduction with AdV.** bmDC were transduced with the indicated AdV types or stimulated with 10 µg/ml or 100 µg/ml LPS. After 24 hours, the supernatants were collected and analyzed for the presence of the indicated cytokines. The cytokine concentrations were determined and fold-changes compared to the unstimulated bmDCs were calculated. (A-J) Dot plot representations of the concentration of the indicated cytokines in the supernatant of bmDCs after the indicated stimulations.

Each dot indicates an individual sample, bars indicate mean values, whiskers indicate the standard deviation. Data were acquired in at least two independent experiments. The dashed line indicates the mean concentration of the indicated cytokine in the supernatant of unstimulated bmDCs, * indicates a statistically significant difference compared to the unstimulated control ($P < 0.05$, One-Way ANOVA).

Supplementary Figure S3

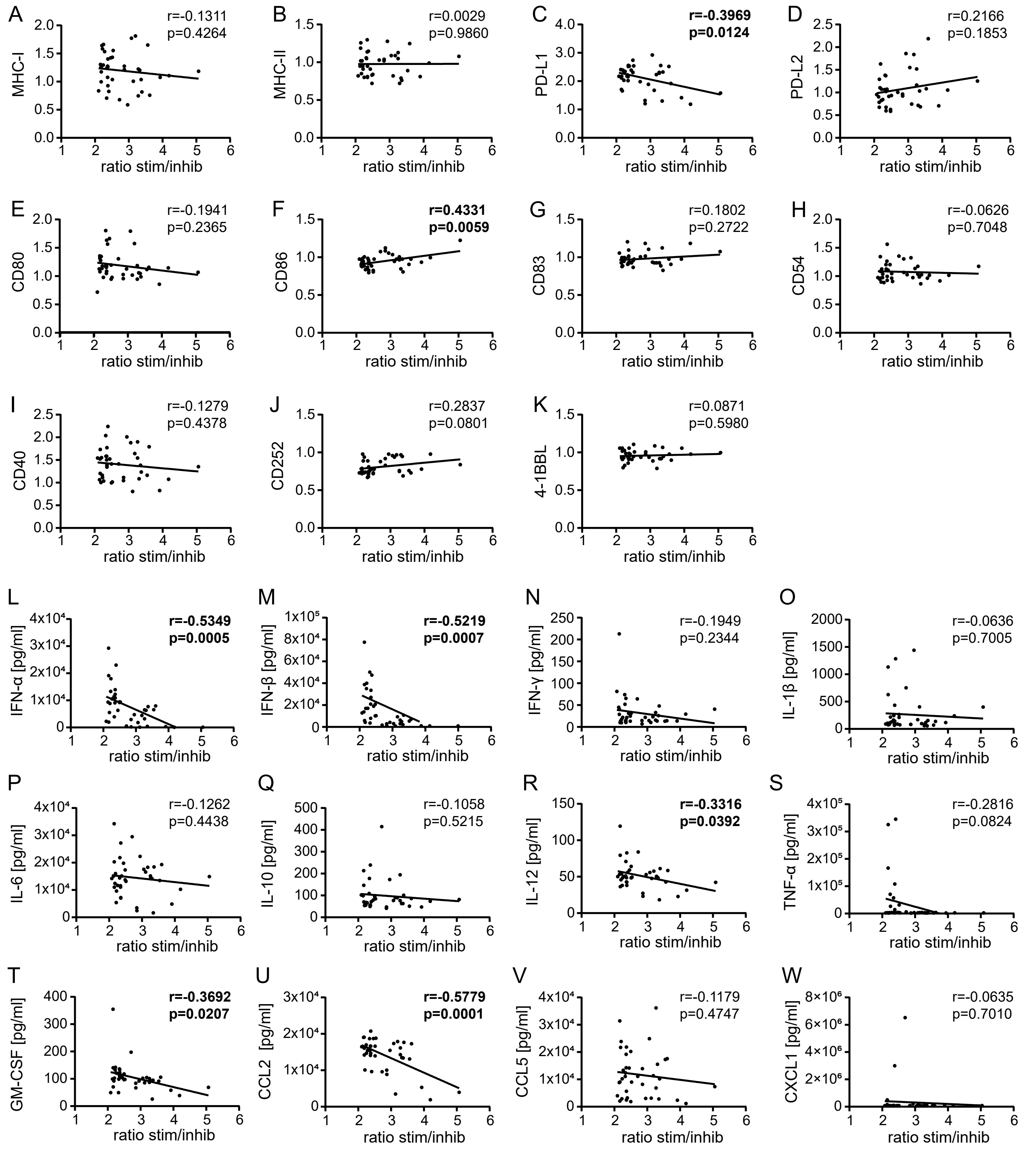


**Correlation analysis of the ratio of HAdV stimulatory and inhibitory TLR9 motifs with expression levels of DC surface markers and cytokine secretion levels.** The HAdV genomic content of stimulatory (aacgtt, gacggt, gacgtt, gccggt, gtcgct, gtcggt, gtcgtt, gggggggg) and inhibitory (ccnddnnggg, ttaggg) TLR9 motifs was analyzed for all 39 HAdV types analyzed in our study, and the ratio of stimulatory and inhibitory motifs was calculated and used to perform Pearson correlation analyses with the expression levels of DC surface markers as shown in Figure 4 (A-K) and with the secretion levels of cytokines by DCs as shown in Figure 5 (L-W).
r, Pearson correlation coefficient; statistically significant correlations are indicated by bold type ($P$ < 0.05).
